# Supplementary material for: A set of multi-entry identification keys to African frugivorous flies (Diptera, Tephritidae)
Source: Zookeys. 2014 Jul 24;(428):97–108. doi: 10.3897/zookeys.428.7366 (PMC4143993; doi:10.3897/zookeys.428.7366)
Supplement: Supplementary material 6 — Key to Ceratitis [file zookeys-428-097-s006.zip › SF6_ZooKeys_key to Ceratitis/key/SF6_ZooKeys_key to Ceratitis/Media/Html/Ceratitis pinax.htm]

Ceratitis (Ceratitis) pinax Munro


***Ceratitis*** **(*Ceratitis*) *pinax*
Munro**

*Ceratitis* (*Pinacochaeta*)  *pinax*
Munro, 1933: 34.

Body
length: 3.96 (3.65-4.45) mm; wing length: 4.11 (3.90-4.45) mm.

Male

Head.
Antenna yellow. Third antennal segment twice as long as second segment. Arista
with short hairs. Frons yellow, sometimes with two whitish but indistinct spots
near antennal implant; with short scattered hairs which are largely the same
colour as frons. Frontal, ocellar and orbital bristles black; lower frontal
weakly developed; lower orbital modified, ribbon shaped, black and slightly
shorter than arista, tip variable usually blunt, sometimes pointed, rarely
flattened and slightly widened. Face yellow-white, lower eye margin with
slightly darker colouration. Genal bristle and setulae reddish, strongly
developed. Postocellar and outer vertical pale.

Thorax.
Postpronotum white, spot more orange-brown, not black, sometimes indistinct.
Mesonotal pattern: ground colour orange-brown, microtrichiae pattern silvery
with brownish shine, spots brown-black, sutural yellow-white spots present but
sometimes indistinct, prescutellar yellow-white markings merged. Scapular setae
pale. Scutellum yellow-white, basally with two dark separate spots, apically
with three merged spots, usually incised to halfway. Anepisternum with lower
half darker yellow, dark pilosity in lower half. 

Legs
yellow; setation typical for subgenus, mainly pale especially on femora. Fore
femur posteriorly with bush of longer dark reddish hairs along entire length,
basally usually darker and almost black; posterodorsal hairs longer; short
anteroventral hairs and ventral spines black. Hind femur with longer hairs
dorsally and ventrally on apical fourth. 

Wing.
Banding; marginal continuous, sometimes with incomplete interruption; cubital
band usually free, rarely joined with discal band; medial band absent;
crossvein r-m at middle of discal cell. Vein R1 before or equal with crossvein
r-m. Crossvein dm-cu posteriorly more outwards than anteriorly.

Abdomen.
Yellow. Setation and banding typical for subgenus.

Female

As
in male except for the following characters: Antenna yellow-orange; third
antennal segment two to three times longer than second segment.  Orbitals not
modified. Both frontal bristles well developed. Genal bristles dark.
Anepisternum without dark pilosity. Crossvein r-m at or just beyond middle of
discal cell. Crosvein dm-cu posteriorly more inwards than anteriorly. Vein R1
equal with crossvein r-m. Oviscape shorter than abdominal terga.

(Description
after De Meyer & Freidberg, in press)
